# Evaluation of Photon Interaction Parameters of Some Antioxidants for Food Irradiation Applications

**DOI:** 10.3390/ma15186376

**Published:** 2022-09-14

**Authors:** Arzu Kavaz Yüksel, Hesham M. H. Zakaly, Antoaneta Ene

**Affiliations:** 1 Department of Food Technology, Technical Sciences Vocational School, Atatürk University, Erzurum 25240, Turkey; 2 Physics Department, Faculty of Science, Al-Azhar University, Assiut Branch, Assiut 71524, Egypt; 3 Institute of Physics and Technology, Ural Federal University, 620002 Yekaterinburg, Russia; 4 INPOLDE Research Center, Department of Chemistry, Physics and Environment, Faculty of Sciences and Environment, Dunarea de Jos University of Galati, 47 Domneasca Street, 800008 Galati, Romania

**Keywords:** antioxidant, gamma radiation, food irradiation

## Abstract

This study aimed to investigate the interaction parameters of antioxidant molecules in some spices and vegetables with gamma radiation. At first, mass attenuation coefficients (MACs, cm^2^/g) of gingerol, rosmarinic acid, quercetin, curcumin, eugenol, piperine, allicin, and capsaicin molecules were determined at the photon energies (13–1332 keV) emitted from the radioactive isotopes Am-241, Ba-133, Co-60, and Cs-137 with the help of the EpiXS and WinXCOM programs. The smallest and largest MAC values were found as 1.20 and 8.48 cm^2^/g at 13 keV and 0.059 and 0.058 cm^2^/g at 1332 keV for eugenol and allicin, respectively. It was observed that both results support each other. Using the MAC values, the effective atomic number and electron density (Z_eff_ and N_eff_) values of the molecules were derived. The Z_eff_ values for gingerol and allicin were obtained in the range of 5.79–3.40 and 13.85–4.53, respectively. The variation of the buildup factors of antioxidants in the range of 0.015–15 MeV depending on the chemical composition and penetration depth were also examined. It was noticed that the photon accumulation was the lowest in allicin and the highest in gingerol and eugenol. The results obtained from this study will make an essential contribution to dose calculations in food irradiation studies.

## 1. Introduction

The food irradiation process involves the process of exposing food materials to a source of energy capable of stripping electrons from individual atoms in the targeted food material. This irradiation method of food preservation can be considered a more advanced form of food preservation. Food irradiation has been approved by the World Health Organization (WHO) and is currently being applied in over 40 countries, and approximately 500,000 tons of food products have been irradiated yearly in the world [1]. The general sources of gamma rays used for food irradiation purposes are those that are produced from radioactive substances. The applied sources for foods are generally cesium-137 (^137^Cs) and cobalt-60 (^60^Co; the most common). They have 1.17 and 1.33 MeV (^60^Co) and 0.662 MeV (^137^Cs) energy levels [2]. Both cobalt-60 and cesium-137 spread highly penetrating gamma rays that can be used in food or packaging material [3]. The basic unit for measuring irradiation is the gray (Gy), which is the amount of irradiation energy in 1 kg of food. The amount of irradiation used for food products is carefully controlled by regulatory authorities. The irradiation dose used for a food product depends on the degree of perishability, the food composition, and the intensity of potentially harmful microorganisms.

Spices with aromatic properties are mostly produced outdoors and dried outside under the sun. After this process, spices become highly contaminated. Spices and herbs used extensively in many foods can put consumer health at risk [4,5,6,7,8,9]. Food irradiation (on red and white meats, spices, and some solid foods) can be used to inactivate pathogenic bacteria that cause food spoilage. Eggs and larvae of insects can be killed in fresh vegetables and fruits by food irradiation [10,11]. There is no change in the sensory properties and quality of irradiated foods with this process. For this purpose, irradiation is the most widespread application on a commercial scale [12,13].

Piggott and Othman exposed black pepper samples to gamma rays at doses of 10, 20, and 30 kGy, and the essential oil content of the samples was determined. The essential oil content did not change with the increase in the radiation dose [14]. The use of gamma radiation for the decontamination of spices, herbs, and some solid foods has been approved by the Codex Alimentarius Commission (CDC). As reported in many studies, the adverse effects of ionizing radiation in food depend on the dose applied, and, in most cases, they can be ignored. Gamma rays or X-rays of up to 5 MeV and electrons of up to 10 MeV energies can be used in food irradiation, and doses of up to 10 kGy are allowed. The sensory properties of most spices are well preserved between 7.5 and 15 kGy [15,16]. Karadaş et al. [17] examined the physicochemical properties of sumac fruit by irradiating them at 2.5, 5, 7.5, and 10 kGy doses. From the results obtained, it was reported that ionizing radiation did not have a negative effect on the quality of sumac oil and a dose of 5 kGy would be sufficient. Studies have shown that the negative effects of food irradiation are negligible and can be applied to many food groups. From this point of view, it would be quite remarkable to examine the relationship of active substances in various vegetables and spices with radiation.

This study investigated the gamma-ray photon interaction parameters of molecules with antioxidant properties in some spices and vegetables. The mass attenuation coefficient, effective atomic number and electron density, energy absorption, and exposure buildup factors of gingerol, rosmarinic acid, quercetin, curcumin, eugenol, piperine, allicin, and capsaicin molecules were extensively investigated. We believe that the data obtained from this study will contribute to food irradiation applications.

## 2. Material and Method

In this study, the photon interaction parameters of the active ingredients of the commonly used spices were studied. The names and chemical formulas of the ingredients are given in Table 1. The degree of attenuation of the incoming radiation of the sample was determined by the Lambert–Beer law [18,19];

(1) $$\mu =ln(I_{0}/I)/x$$

*I* and *I*_0_ denote the incoming and transmitted photon intensities, respectively, *x* refers to the thickness of the sample, and μ is the linear attenuation coefficient. To eliminate the dependence of the linear attenuation coefficient on the absorbent material density, one may use the mass attenuation coefficient of the material instead of its linear counterpart. Mass attenuation coefficient ($\mu_{\rho }$ MAC, cm^2^/g) represents the amount of radiation absorbed per unit area per unit mass and is calculated by a ratio between μ and the density of the sample (*ρ*) [20]. In order to compute the radiation interaction parameters of the active molecules, first of all, the MAC values of the molecules were generated using the mixture method (Equation (1)) with the EpiXS [21] and WinXCOM [22] programs.

(2) $$\mu_{\rho }=\underset{i}{\sum }w_{i}{\left(\mu /\rho \right)}_{i}$$

XCOM is an interface for determining cross sections and attenuation coefficients in a standard energy range (1 keV–100 GeV) or selected energies. It also presents the cross sections of events such as incoherent, coherent, photoelectric effect, and pair formation. It performs the calculation of cross sections and reduction coefficients for all elements in the periodic table, as well as for compounds and mixtures. The Windows version of this interface was used as the WinXCOM program.

The most recent source of photon cross sections is EPICS2017 [23]. Existing master codes such as MCNP, Geant4, FLUKA, and PHITS used to evaluate MACs [24,25,26] are currently based on data replacing EPICS2017, which is designed to replace embedded data in Monte Carlo codes.

EpiXS [21] is an alternative software released similar to WinXCOM. It has new coupling energies, absorption edge energies, and partial and total cross sections compared to its EPICS2017 counterparts.

In mixed materials, *Z_eff_* which is called the effective atomic number takes a value between the smallest and largest of the atomic numbers of the elements in the substance and calculated utilizing the following relation [27,28,29]:

(3) $$Z_{eff}=\frac{\sum_{i}f_{i}A_{i}{\left(\frac{\mu }{\rho }\right)}_{i}}{\sum_{j}\frac{A_{j}}{Z_{j}}{\left(\frac{\mu }{\rho }\right)}_{j}}$$

where *f_i_* is the fractional abundance, and *A_i_* is the atomic weight. The effective electron density (*N_el_*_,_ electron per unit mass of the absorber) is given by the expression of [30,31,32]:

(4) $$N_{el}=N_{A}\frac{Z_{eff}}{\langle A\rangle }\left(\mathrm{electrons}/g\right)$$

As gamma rays interact with matter through Compton scattering, the incident photon energy decreases and changes direction, resulting in scattered secondary photons that can be predicted by the buildup factor. The gamma-ray buildup factor is a coefficient employed to acquire the uncorrected outcome by including the addition of scattered photons. This factor is the ratio of the total detector response to the number of photons that have not undergone any collisions [33,34,35,36]. There are two types of buildup factors: energy absorption (EABFs) and exposure (EBFs) buildup factors. While EABFs are concerned with the amount of energy stored or absorbed in matter interacting with the photon, in EBFs, the quantity of interest is exposure to incident photons [37,38,39]. In this study, the buildup factors of molecules were calculated via the geometric progression (GP) fitting approach using the EpiXS program.

## 3. Results and Discussion

**Figure 1:**
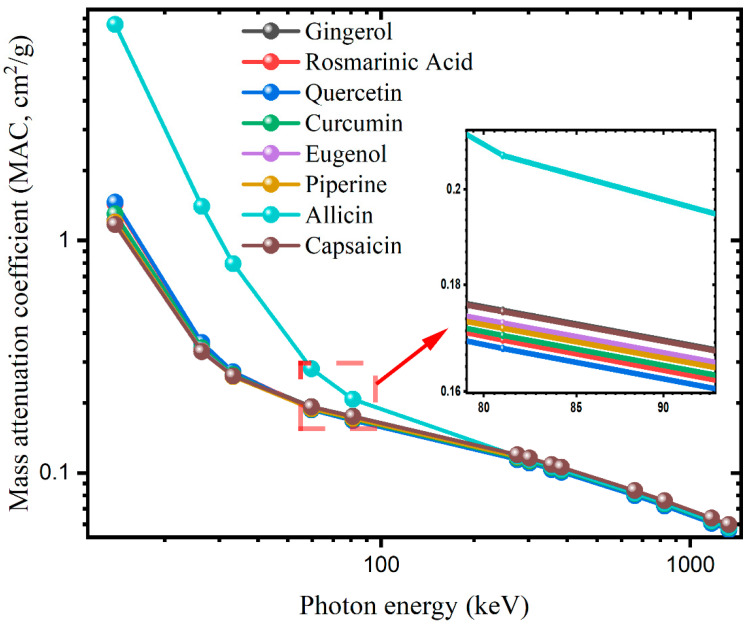
Mass attenuation coefficients (cm^2^/g) of the antioxidants versus the photon energy.

The photon interaction parameters of the active ingredients of some spices and vegetables listed in Table 1 were calculated in the 13–1332 keV energy ranges. The photon energies emitted from Co-60 and Cs-137 isotopes used especially in food irradiation were also included. The mass attenuation coefficients (MACs) obtained with EpiXS and WinXCOM software for the antioxidants are summarized in Table 2. Moreover, in Figure 1, the variation of MAC values with gamma photon energy is plotted. From these results, it is seen that the MAC values are maximum at 13 keV and decrease rapidly up to 100 keV with increasing photon energy. Since the photoelectric absorption (PEA) cross section is proportional to Z^4−5^ in the low-energy region [40], the dependence of MACs on the chemical composition of the sample is high. Eugenol and allicin had the smallest and largest MAC values, respectively, with MACs of 1.20 and 8.48 cm^2^/g at 13 keV and 0.059 and 0.058 cm^2^/g at 1332 keV. In this result, the presence of the S element with a high atomic number in the content of allicin is effective. Since Compton scattering and pair production are effective in the range of 100 keV–1332 keV, MAC values are close to each other in the middle energy region where secondary scatterings increase. In the photon energy range of 1 MeV–1.33 MeV, the MAC values of molecules such as allicin, which contain a heavier element (S), have decreased due to the buildup of the photons in the sample and causing more secondary scatterings.

**Figure 2:**
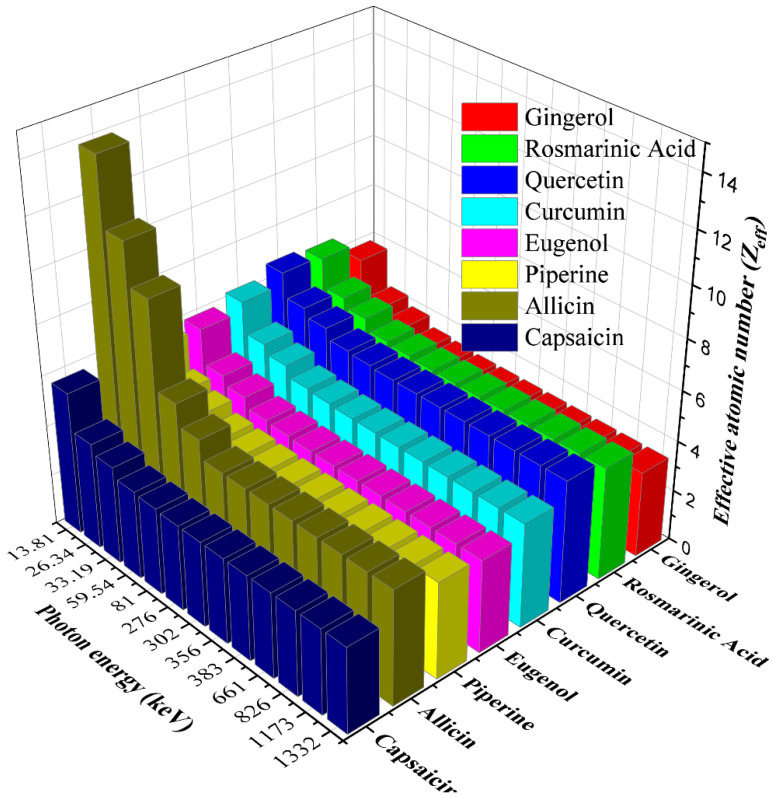
The variation of effective atomic number for the antioxidants with the photon energy.

**Figure 3:**
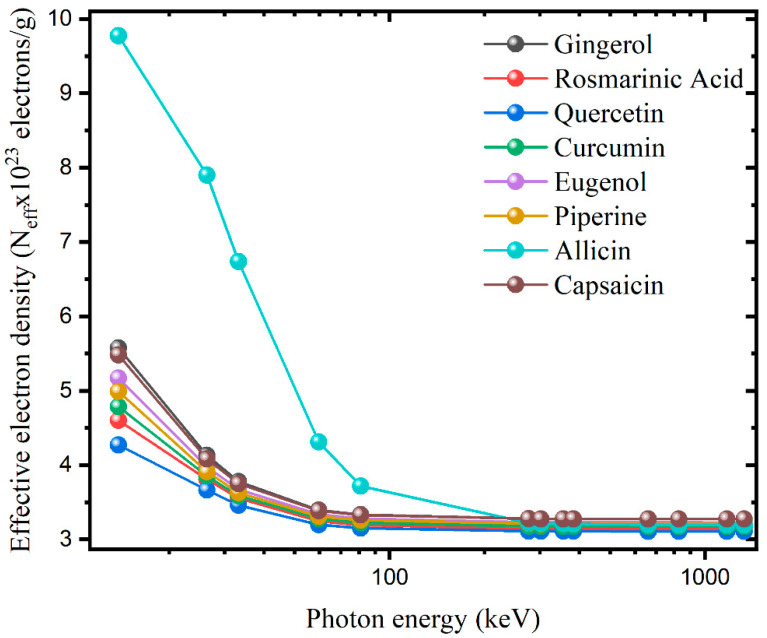
The variation of effective electron density for the antioxidants with the photon energy.

Figure 2 presents the variation of the effective atomic numbers (Z_eff_) of the studied antioxidants with photon energy. As seen in Figure 2, the Z_eff_ values for all samples decrease with increasing photon energy. This can be attributed to the fact that gamma rays interact with matter in three different ways, all of which depend on photon energy. The Z_eff_ values of gingerol, rosmarinic acid, quercetin, curcumin, eugenol, piperine, allicin, and capsaicin molecules are in the range of 5.79–3.40, 6.55–4.47, 6.69–4.87, 6.22–4.12, 5.87–3.66, 5.90–3.80, 13.85–4.53, and 5.67–3.38, respectively. Allicin obtained the highest Z_eff_ values, while the smallest Z_eff_s belong to eugenol and gingerol. The variation of N_eff_ values with photon energy is parallel to Z_eff_ (Figure 3). However, the fact that the average atomic weight is different in each molecule causes some changes. Allicin again owns the largest N_eff_ values, while the N_eff_ values for quercetin are found the lowest. These results show that the Zeff values vary depending on the elements contained in the molecule and are smaller in samples with higher hydrogen content and larger in molecules containing elements with higher atomic numbers.

**Figure 4:**
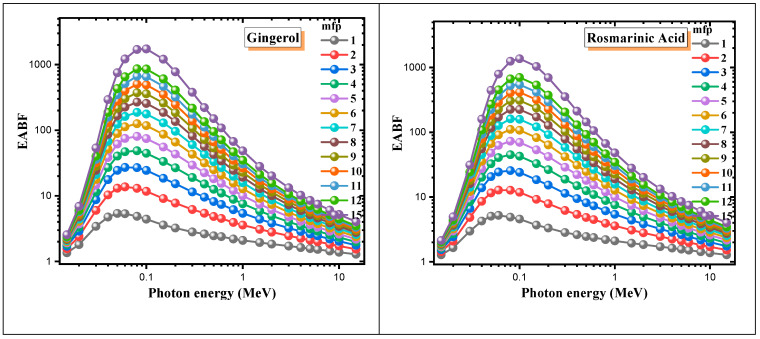

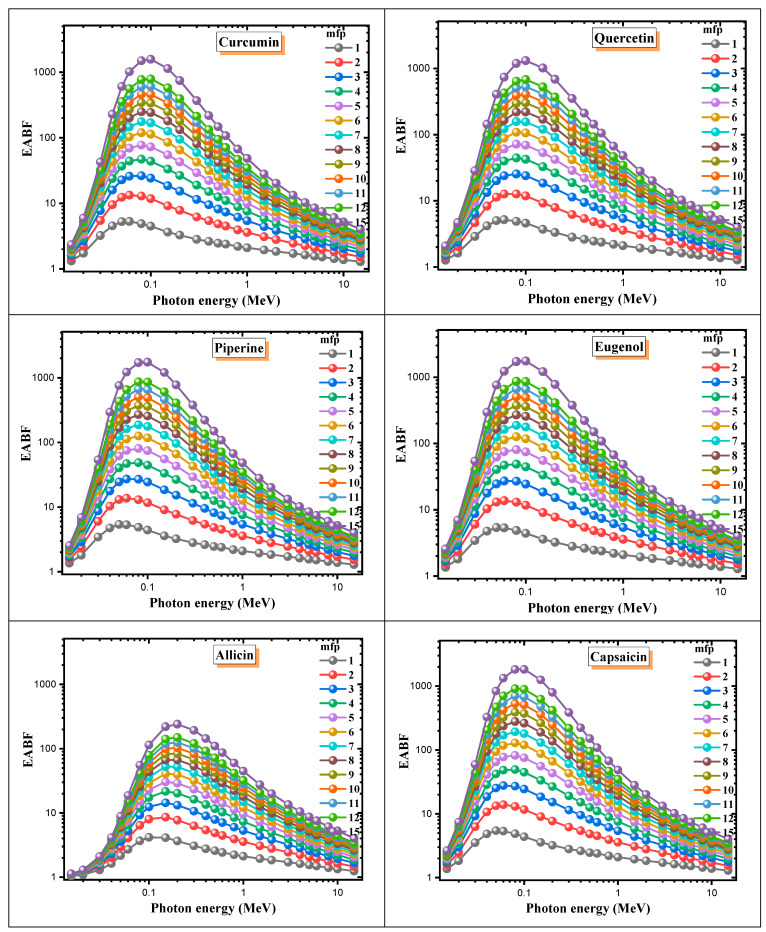
EABF values of the antioxidants for different penetration depths in the 0.015–15 MeV photon energy range.

**Figure 5:**
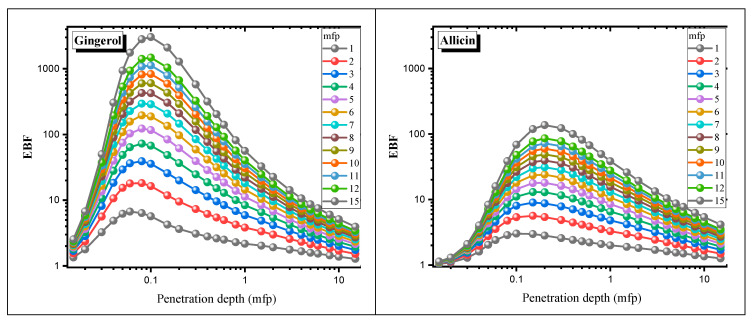
EBF values of the antioxidants for different penetration depths in the 0.015–15 MeV photon energy range.

The energy absorption and exposure buildup factors (EABFs and EBFs) of antioxidant molecules are demonstrated in Figure 4 and Figure 5 in the range of 0.015–15 MeV depending on the gamma photon energy. The EABFs and EBFs for all molecules in the low-energy region take minimum values. The reason the buildup factors are around 1 in this region is that most of their photons are absorbed in the PEA process. At medium energies where the Compton scattering process begins to dominate, the EABFs and EBFs rise rapidly and reach their maximum values at about 0.1 MeV.

**Figure 6:**
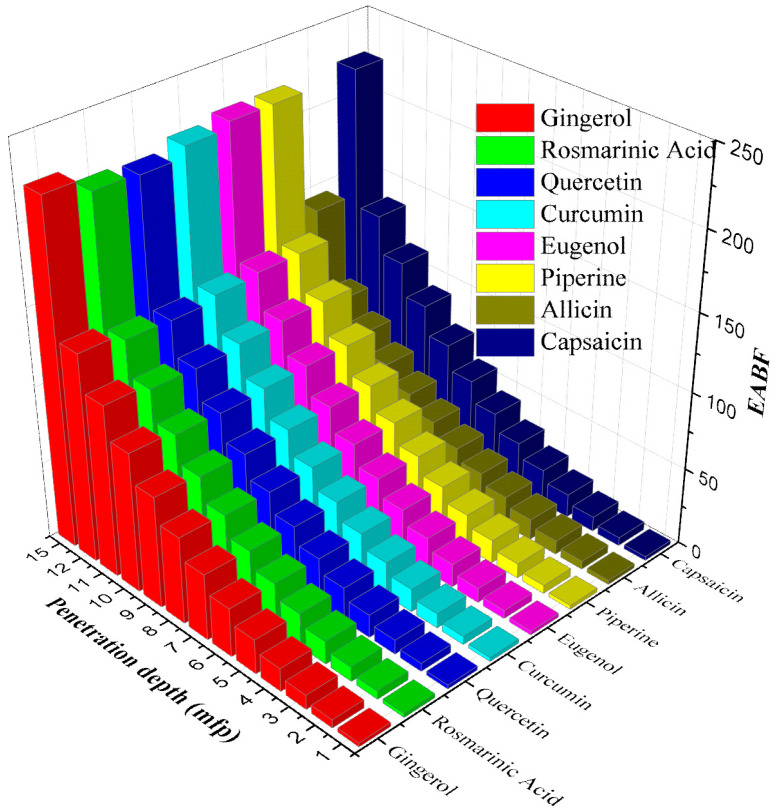
EABF values of the antioxidants versus the different penetration depths and chemical compositions at 0.4 MeV.

**Figure 7:**
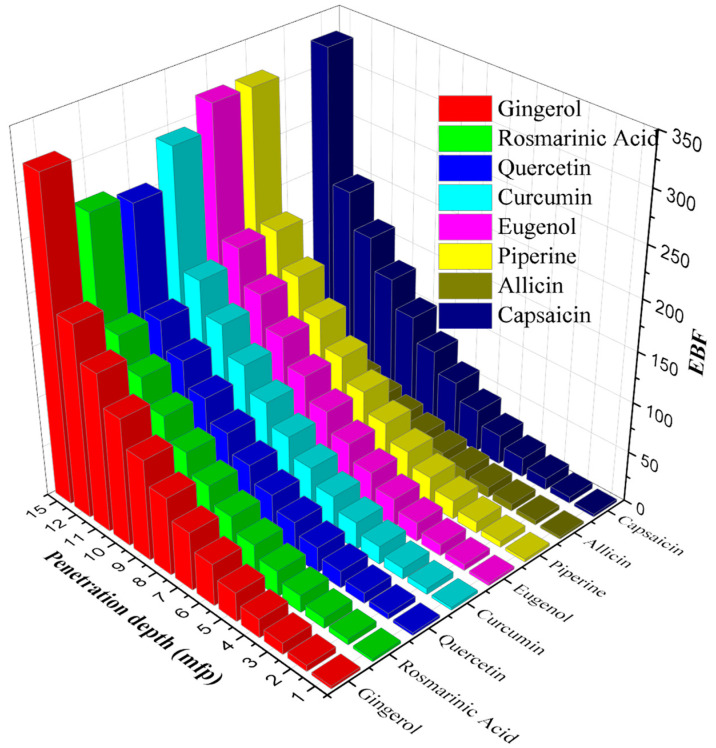
EBF values of the antioxidants versus the different penetration depths and chemical compositions at 0.4 MeV.

After this point, the buildup factors start to decrease rapidly. In the 1–15 MeV photon energy range, where the pair production process occurs, the buildup coefficients approach 1 again, as the photons annihilate at low energies. It is clear from Figure 4 and Figure 5 that allicin has significantly smaller EABF and EBF values than other antioxidants. It is noteworthy that the photon buildup is higher in gingerol and eugenol molecules compared to the others. It is also seen that both buildup coefficients enhance significantly with increasing penetration depth. Moreover, in Figure 6 and Figure 7**,** the variation of EABF and EBF values depending on the penetration depth and chemical composition is presented for a constant photon energy of 0.4 MeV. It is significant that EABFs and EBFs are strongly dependent on the chemical composition of the molecules and the depth of penetration. It is clear that photon buildup increases with enhancing sample thickness. Allicin, which contains the heavier S element, has high Z_eff_ values, and the buildup coefficients are the smallest for this molecule. Gingerol and capsaicin have the largest buildup factor values of 0.4 MeV, corresponding to medium energy. Another detail worth noting from Figure 6 and Figure 7 is that the EABF values are lower than the EBFs. This indicates that the photon buildup in the antioxidants is lower than in the air.

## 4. Conclusions

In the current work, we examined the degree of interaction of antioxidants in some spices and vegetables with gamma-ray photons. In recent years, it has become a very common method to extend the shelf life of foods by irradiating them with gamma rays. It will be very meaningful to examine the interaction of the active ingredients of foods with photons during this irradiation. For this purpose, we evaluated the photon interaction parameters of eight antioxidants in the 13–1332 keV energy range. The MAC and Zeff values were found to be 8.4912–0.0584 cm^2^/g and 13.85–4.53 for allicin and 1.2029–0.0590 cm^2^/g and 5.87–3.66 for eugenol, respectively, in the energy range studied. It was determined that the highest MAC, Z_eff_, and N_eff_ values belonged to allicin (garlic), and the smallest ones belonged to eugenol and gingerol (clove and ginger). The highest values of EABFs and EBFs are found for gingerol, while allicin absorbs the incoming photons the most and inhibits the buildup of photons more than the others. Correct dose selection and irradiation conditions are extremely important in food irradiation. Examining the interaction parameters of the active ingredients of spices, which are dried foods, with gamma radiation will be useful for dose calculations in food irradiation applications.

## Figures and Tables

**Table 1:** Spice, active molecule names, and chemical formula of the molecules.

Spice Name	Active Molecule Name	Chemical Formula	Molecular Structures
Ginger	Gingerol	C_17_H_26_O_4_	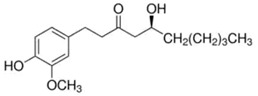
Rosemary	Rosmarinic Acid	C_18_H_16_O_8_	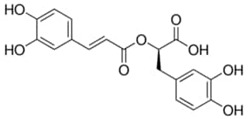
Onion	Quercetin	C_15_H_10_O_7_	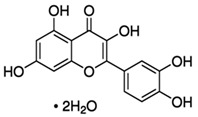
Turmeric	Curcumin	C_21_H_20_O_6_	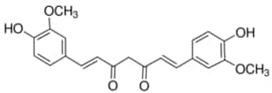
Cloves	Eugenol	C_10_H_12_O_2_	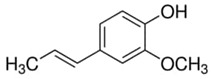
Black Pepper	Piperine	C_17_H_19_ NO_3_	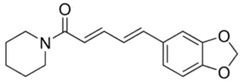
Garlic	Allicin	C_6_H_10_OS_2_	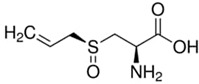
Red Pepper	Capsaicin	C_18_H_27_NO_3_	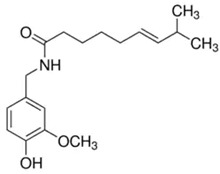

**Table 2:** The mass attenuation coefficient values (cm^2^/g) obtained by using EpiXS and WinXCOM programs for the antioxidant molecules.

**Photon Energy** **(keV)**	**Gingerol**		**Rosmarinic Acid**		**Quercetin**		**Curcumin**	
	**EpiXS**	**WinXCOM**	**EpiXS**	**WinXCOM**	**EpiXS**	**WinXCOM**	**EpiXS**	**WinXCOM**
13.81	1.2225	1.2234	1.4324	1.4336	1.4597	1.4609	1.3008	1.3018
26.34	0.3394	0.3398	0.3614	0.3619	0.3634	0.3639	0.3449	0.3454
33.19	0.2649	0.2651	0.2720	0.2722	0.2720	0.2722	0.2647	0.2649
59.54	0.1926	0.1926	0.1881	0.1881	0.1866	0.1866	0.1881	0.1881
81	0.1750	0.1750	0.1694	0.1694	0.1678	0.1678	0.1703	0.1702
276	0.1196	0.1195	0.1149	0.1148	0.1137	0.1136	0.1160	0.1159
302	0.1157	0.1157	0.1112	0.1111	0.1100	0.1100	0.1122	0.1121
356	0.1087	0.1086	0.1044	0.1043	0.1033	0.1032	0.1054	0.1052
383	0.1057	0.1056	0.1015	0.1015	0.1004	0.1004	0.1024	0.1024
661	0.0840	0.0839	0.0806	0.0806	0.0798	0.0797	0.0814	0.0813
826	0.0758	0.0758	0.0728	0.0728	0.0720	0.0720	0.0735	0.0734
1173	0.0640	0.0638	0.0614	0.0613	0.0607	0.0606	0.0620	0.0619
1332	0.0599	0.0598	0.0575	0.0574	0.0569	0.0568	0.0581	0.0580
**Photon Energy** **(keV)**	**Eugenol**		**Piperine**		**Allicin**		**Capsaicin**	
	**EpiXS**	**WinXCOM**	**EpiXS**	**WinXCOM**	**EpiXS**	**WinXCOM**	**EpiXS**	**WinXCOM**
13.81	1.2020	1.2029	1.1978	1.1988	8.4826	8.4912	1.1679	1.1688
26.34	0.3343	0.3347	0.3327	0.3331	1.4016	1.4037	0.3319	0.3324
33.19	0.2611	0.2613	0.2597	0.2599	0.7921	0.7934	0.2612	0.2614
59.54	0.1899	0.1899	0.1888	0.1888	0.2797	0.2801	0.1920	0.1919
81	0.1726	0.1726	0.1716	0.1716	0.2074	0.2076	0.1748	0.1747
276	0.1180	0.1179	0.1173	0.1172	0.1179	0.1178	0.1196	0.1195
302	0.1141	0.1141	0.1134	0.1134	0.1139	0.1138	0.1157	0.1157
356	0.1072	0.1071	0.1066	0.1064	0.1067	0.1066	0.1087	0.1086
383	0.1042	0.1042	0.1036	0.1035	0.1036	0.1036	0.1057	0.1056
661	0.0828	0.0827	0.0823	0.0823	0.0821	0.0820	0.0840	0.0839
826	0.0748	0.0747	0.0743	0.0743	0.0741	0.0740	0.0758	0.0758
1173	0.0631	0.0630	0.0627	0.0626	0.0624	0.0623	0.0640	0.0638
1332	0.0591	0.0590	0.0587	0.0586	0.0585	0.0584	0.0599	0.0598

## Data Availability

The data presented in this study are available on request from the corresponding author.
